# Minimum requirements for motility of a processive motor protein

**DOI:** 10.1371/journal.pone.0185948

**Published:** 2017-10-10

**Authors:** Andreja Šarlah, Andrej Vilfan

**Affiliations:** 1 Faculty of Mathematics and Physics, University of Ljubljana, Ljubljana, Slovenia; 2 J. Stefan Institute, Ljubljana, Slovenia; University of Minnesota Twin Cities, UNITED STATES

## Abstract

Motor proteins generally have a two-way coupling between the ATP hydrolysis site, the lever movement and the binding affinity for their track, which allows them to perform efficient stepping. Here we explore the minimal requirements for directed motility based on simpler schemes in which the binding/unbinding from the track is decoupled from the ATPase cycle. We show that a directed power stroke alone is not sufficient for motility, but combined with an asymmetry in force-induced unbinding rates it can generate stepping. The energetic efficiency of such stepping is limited to approximately 20%. We conclude that the allosteric coupling between the ATP hydrolysis and the track binding is not strictly necessary for motility, but it greatly improves its efficiency.

## Introduction

Linear motor proteins convert the free energy gained from the hydrolysis of ATP into mechanical work while moving along their tracks [1–4]. This is done by an ATP hydrolysis with subsequent multi-stage product release. During the cycle a motor binds to the track, undergoes a conformational change (power stroke), releases the track and returns to the original conformation (recovery stroke). The nucleotide state is coupled in different ways to the power stroke and to the binding affinity for the track (actin or microtubule). For example, in the Lymn-Taylor cycle that describes the activity of myosin, the power stroke is coupled to phosphate release and the recovery stroke to ATP hydrolysis. Myosin’s actin binding affinity is strongly reduced by the binding of an ATP molecule and increased after ATP hydrolysis [5].

The two-way coupling between the catalytic site, track binding and lever motion is a universal feature of myosins, kinesins and dyneins, despite the diversity among their structures, types of tracks, directionality and kinetics. This raises the question whether a motor can be functional with a less complex mechanism and to what extent this would impair its efficiency.

An early model for kinesin assumed that ATP hydrolysis only affects the tension between kinesin’s two heads, whereas the detachment from the microtubule is only caused by the mechanical load on a head [6]. The asymmetry of potential wells that describe the interaction of a head with the track, along with broken time-reversal symmetry, was thus central for the establishment of directional motility, which, however, followed a (later refuted) inchworm pattern. An alternative kinesin model proposed tight coupling between the chemical state and the microtubule binding, but included only minimal strain dependence of the kinetic rates [7].

Dynein (reviewed in [8–10]) is special among motor proteins due to the large spatial separation between its ATP hydrolysis sites (on the ring) and both (i) the microtubule binding domain (connected to the ring through a coiled-coil stalk) and (ii) the tail via the linker whose docking to the ring acts as a power stroke [11]. The communication through the stalk is mediated by the relative sliding of the two *α*-helices forming the coiled coil [12–15]. It modulates the microtubule binding affinity depending on the nucleotide in the primary catalytic site [16], but it also affects nucleotide release from the catalytic site depending on the attachment/detachment of the motor to the microtubule [17, 18]. On the other side the hydrolysis cycle is coupled to the linker swing [11, 19]. Although allosteric interaction through the coiled-coil stalk appears to be a crucial part of dynein’s mechanism, experiments show that directed motility is also possible in hetero-dimers with one head completely lacking the ring [20]. Similar results were obtained in kinesin dimers in which one head is occupied by non-hydrolyzable AMP-PNP [21] or inactivated [22]. Under load, dynein can step processively even in the absence of ATP, pointing to the role of directed load-induced unbinding [23].

In this paper we propose a minimal model to study the efficiency of stepping of a dimeric motor without any coordination between its two motor domains and without any interaction between the ATPase site and the track binding domain. The ATP hydrolysis is, however, tightly coupled to the linker movement. Our main motivation is to investigate how efficiently dynein could walk without the allosteric communication through the stalk which connects its catalytic site with the microtubule binding domain. However, most of the discussion is general and applies to any dimeric motor with a 2-state cycle whose binding affinity for the track is not directly coupled to its chemical state. We show that an asymmetric force-dependence of unbinding rates, together with an intramolecular tension caused by ATPase-dependent linker movement is sufficient for directed motility. We discuss the influence of kinetic rates on the stepping efficiency and determine the optimal working regime of such motor. The results allow us to estimate how much efficiency is gained by the allosteric interaction through dynein’s stalk, but they also serve as guidance for designing synthetic bipedal walkers [24, 25].

## Model

### Elastic model

**Fig 1:**
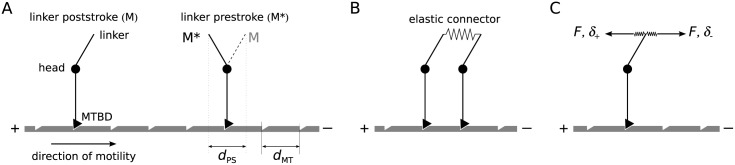
Geometry of the model. (A) A model monomeric motor with the linker in two possible positions, post-powerstroke (*M*) and pre-powerstroke (M*). The end of the linker moves for *d*_PS_ between the two positions. (B) In a dimeric motor the ends of the linkers are coupled through an elastic connector. (C) Asymmetric unbinding rates. The distance parameter (*δ*_+_, *δ*_−_) depends on the direction of the force acting on the bond.

We discuss the stepping of a dimeric motor moving along a one-dimensional track (microtubule—MT in what follows) with discrete binding sites uniformly spaced at distance *d*_MT_, as shown in Fig 1A. Each motor head can be bound to the track (states *M*/M*) or free (states *m*/m*). Here M*/m* denote the pre-powerstroke and *M*/*m* the post-powerstroke states. The linker, which connects a head to its partner head and to the cargo, acts as a swinging lever arm and moves by distance *d*_PS_ during the power stroke. We introduce an elastic connector joining the two linkers of the dimer (Fig 1B), effectively representing all compliant elements in the dimer (in dynein, the linkers were identified as the main source of flexibility [26]).

In the one-dimensional model we only consider the longitudinal components of the forces—which perform work against the applied load—and neglect any effect that off-axis forces could have on the transition rates. For moderate deformations considered here, the connector can be described as a linear spring. If it is stretched by distance *d*, its force (internal tension in the dimer) is *F*_int_ = *Kd* and the elastic energy *U*_int_ = *Kd*^2^/2. For linear springs the internal tension is independent of the applied load *F*. The forces which the lead ($F_{b}^{L}$) and the trail ($F_{b}^{T}$) head exert on the track can be written as

$$\begin{array}{c} F_{b}^{L/T}=F/2\pm F_{\text{int}}. \end{array}$$ (1)

A positive sign means that the force is pulling towards the MT plus end. In a dimer with one bound and one free head the force on the bound head is simply *F*_*b*_ = *F*.

### Kinetic rates

We assume that the detachment rate of a head from the MT depends exponentially on the longitudinal force *F*_*b*_ (Bell’s model [27])

$$\begin{array}{c} k_{-\text{MT}}=k_{-\text{MT}}^{0}{\text{e}}^{\frac{|F_{b}|\delta }{k_{\text{B}}T}}, \end{array}$$ (2)

where $k_{-\text{MT}}^{0}$ is the unloaded unbinding rate and *δ* the distance parameter. The force-induced unbinding can by asymmetric with respect to the direction of the applied force (Fig 1C). We therefore introduce different distance parameters, *δ*_−_ for forces acting towards the MT minus end (*F*_*b*_ < 0) and *δ*_+_ for forces towards the plus end (*F*_*b*_ > 0), and write them as

$$\begin{array}{c} \delta_{\pm }=\bar{\delta }\;\mp \;\delta_{a}. \end{array}$$ (3)

By *k*_+MT_ we denote the binding rate of the second head to a specific site on the track when the first head is already bound. The binding and unbinding rate follow the principle of detailed balance,

$$\begin{array}{c} k_{+\text{MT}}/k_{-\text{MT}}={\text{e}}^{-\Delta G/k_{\text{B}}T}, \end{array}$$ (4)

where Δ*G* = Δ*G*_+MT_ + Δ*U* is the free energy difference between the double-bound and the single bound state. It consists of a binding energy Δ*G*_+MT_ and the mechanical contribution Δ*U* = Δ*U*_int_ + *F*Δ*x* that contains elastic energy and work against the load. With the unbinding rate given by Eq (2), the binding rate has the form

$$\begin{array}{c} k_{+\text{MT}}=k_{+\text{MT}}^{0}{\text{e}}^{\frac{|F_{b}|\delta }{k_{\text{B}}T}}{\text{e}}^{-\frac{\Delta U}{k_{\text{B}}T}}. \end{array}$$ (5)

**Fig 2:**
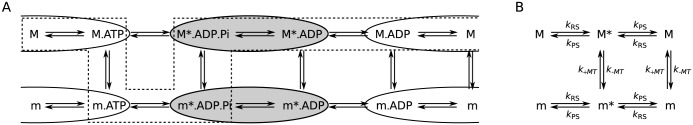
Chemical cycle of a single motor head. (A) The ATP hydrolysis can take place both in a MT-attached (*M*) or MT-detached (*m*) head; the asterisk denotes states with the linker in the pre-powerstroke position and ATP, ADP.Pi, and ADP denote the nucleotides bound to the head. The 5-state ATPase cycle of the dynein motor [16] is enclosed by the dashed frame. The ovals highlight the effective states of the simplified cycle in our model (grey ovals: M* and m*, white ovals: *M* and *m*). (B) The 2-state effective cycle of our model.

Although the ATP hydrolysis cycle of a motor protein is more complex (Fig 2A), we reduce it to two transitions involving linker movement (Fig 2B), i.e., the power stroke (M* → *M*, rate $k_{\text{PS}}^{0}$) and recovery stroke (*M* → M*, rate $k_{\text{RS}}^{0}$). We first neglect the effect of load on these rates—an assumption that is plausible as long as the elastic energies involved are sufficiently smaller than the free energy available from ATP hydrolysis. A model that also includes strain dependent reverse rates and is thus thermodynamically consistent is discussed in section Results and discussion.

### States of the dimeric motor

In the following we assume that the two heads of a dimer can only occupy two adjacent binding sites on the track, separated by *d*_*MT*_. The stepping of kinesin [28] and mammalian dynein [29] is characterized by regular 8-nm steps. However, in yeast dynein a wider distribution of steps has been observed [30, 31]. Allowing the binding to non-adjacent sites would not affect the discussion of minimum motility requirements in our paper and would only have a quantitative effect on the results by increasing the average step length.

**Fig 3:**
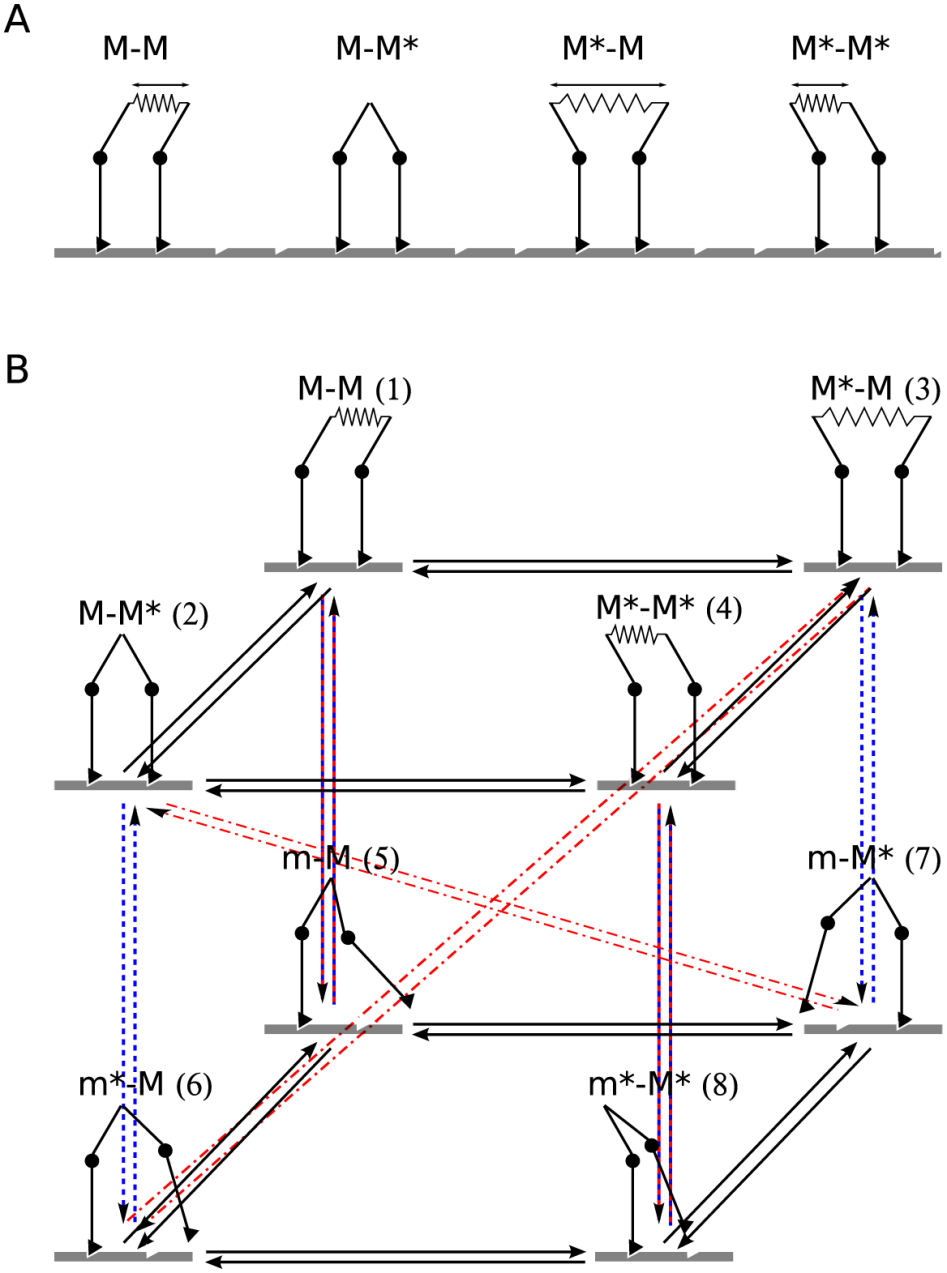
States in the dimeric motor. (A) The bound states. The arrows show the extension of the elastic connector joining the two linkers. (B) Transitions between the states. Lower plane: Transitions between the states with one head unbound (states *m*, m*). Upper plane: Transitions between states with both heads bound. Arrows between the two planes show binding/unbinding of the lead (red) and the trail (blue) head.

The dimer then has 4 mechanical states: *M* − *M*, *M* − M*, M* − *M* and M* − M* (Fig 3A). In this notation the first symbol describes the state of the trail head and the second that of the lead head. Together with 4 states in which one head is detached (denoted *m* − *M*, *m* − M*, m* − *M* and m* − M*) there are in total 8 states for bound dimers, which we enumerate as shown in Fig 3B. The connector extension (Fig 3A) is *d*_1_ = *d*_4_ = *d*_MT_ in the states *M* − *M* and M* − M* and *d*_2,3_ = *d*_MT_ ∓ *d*_PS_ in the states *M* − M* and M* − *M*. The resulting network has 2 × 14 transitions. A similar formulation has been used in models of kinesin [32], myosin V [33], and also dynein [34].

### Parameters for the dynein motor

We now introduce the specific set of parameters for the dynein motor. According to crystallographic [18, 35] and electron microscopic [11, 19] studies the linker swing distance approximately matches one MT period. Thus, we set *d*_PS_ = *d*_MT_, which gives *d*_2_ = 0 and *d*_3_ = 2*d*_MT_. For the connector stiffness we choose a value *K* = 0.3pN/nm which leads to reasonable values for the maximum elastic energy and internal tension, i.e., *U*_3_ = 10*k*_B_*T* and *F*_int,3_ = 5pN.

Single molecule experiments on strongly bound dynein heads show unstrained unbinding rates of ∼1s^−1^ [20, 36]. The effect of applied force is pronouncedly asymmetric. Resisting (MT plus end directed) forces have little effect on the unbinding rate (although some effect has been reported for forces below 2pN [36]). Assisting (minus end directed) forces strongly accelerate unbinding with a distance parameter in the range of 4 − 12nm (“slip bond”). In the following, however, we follow a more general approach and discuss the effect of asymmetric unbinding rates on the stepping efficiency in a broader parameter range.

## Results and discussion

### Analytical solution

We define *c*_*i*_ as the occupancy of each of the 8 dimer states (Fig 3B), normalized such that ∑_*i*_*c*_*i*_ = 1. Their temporal evolution follows the master equation

$$\begin{array}{c} \dot{c}=Mc, \end{array}$$ (6)

with **c** = (*c*_1_, *c*_2_, *c*_3_, *c*_4_, *c*_5_, *c*_6_, *c*_7_, *c*_8_) and *M*_*ij*_ = *K*_*ij*_ − *δ*_*ij*_∑_*l*_*K*_*li*_. Here K is a transition rate matrix with *K*_*ij*_ = *k*_*ij*_ the rate of the transition *i* ← *j*. Explicitly, the transition rate matrix for our model reads

$$\begin{array}{c} K=\left(\begin{array}{cccccccc} 0 & k_{\text{PS}}^{0} & k_{\text{PS}}^{0} & 0 & \;\;\;\;k_{1}^{F}+k_{1}^{B}\;\;\;\; & 0 & 0 & 0\\ k_{\text{RS}}^{0} & 0 & 0 & k_{\text{PS}}^{0} & 0 & k_{2}^{F} & k_{2}^{B} & 0\\ k_{\text{RS}}^{0} & 0 & 0 & k_{\text{PS}}^{0} & 0 & k_{3}^{B} & k_{3}^{F} & 0\\ 0 & k_{\text{RS}}^{0} & k_{\text{RS}}^{0} & 0 & 0 & 0 & 0 & \;\;\;\;k_{4}^{F}+k_{4}^{B}\;\;\\ \;\;k_{1}^{T}+k_{1}^{L}\;\;\;\; & 0 & 0 & 0 & 0 & k_{\text{PS}}^{0} & k_{\text{PS}}^{0} & 0\\ 0 & k_{2}^{L} & k_{3}^{T} & 0 & k_{\text{RS}}^{0} & 0 & 0 & k_{\text{PS}}^{0}\\ 0 & k_{2}^{T} & k_{3}^{L} & 0 & k_{\text{RS}}^{0} & 0 & 0 & k_{\text{PS}}^{0}\\ 0 & 0 & 0 & \;\;\;\;k_{4}^{T}+k_{4}^{L}\;\;\;\; & 0 & k_{\text{RS}}^{0} & k_{\text{RS}}^{0} & 0 \end{array}\right). \end{array}$$ (7)

Here $k_{i}^{F,B}$ is the rate of MT binding into state *i* in the forward or backward direction, respectively, see Eq (5). Similarly, $k_{i}^{L,T}$ is the unbinding rate of the lead or trail head in state *i*, as defined in Eq (2).

In the stationary state $\dot{c}=0$ and the occupancies *c*_*i*_ follow from

$$\begin{array}{c} Mc=0. \end{array}$$ (8)

The average velocity is

$$\begin{array}{c} v=d_{\text{MT}}/2\left(\sum_{i=1}^{4}c_{i}\left(k_{i}^{T}-k_{i}^{L}\right)+\sum_{i=5}^{8}c_{i}\left(k_{i-4}^{F}-k_{i^{\prime }}^{B}\right)\right), \end{array}$$ (9)

where *i*′ = 1, 3, 2, 4 for *i* = 5, 6, 7, 8, respectively. The first term in Eq (9) accounts for the translation of the center of the motor when it unbinds the trail or the lead head from the MT. Similarly, the second term accounts for the translation when the free head binds to the MT in the forward or backward direction.

The ATPase rate per dimer can be formally written as $r_{\text{ATP}}=k_{\text{RS}}^{0}\left(2c_{1}+c_{2}+c_{3}+2c_{5}+c_{6}+c_{7}\right)$. Because we assumed that the ATP hydrolysis, involving transitions *M* → M* → *M*, is independent of the partner head state, the ATPase rate is simply twice (for two heads) that of a cyclic two-step process

$$\begin{array}{c} r_{\text{ATP}}=\frac{2}{1/k_{\text{RS}}^{0}+1/k_{\text{PS}}^{0}}. \end{array}$$ (10)

The ratio between velocity and the ATP hydrolysis rate determines the length of the average translational move per ATP molecule,

$$\begin{array}{c} {\bar{l}}_{\text{ATP}}=\frac{v}{r_{\text{ATP}}}. \end{array}$$ (11)

For coordinated hand-over-hand stepping it could reach ${\bar{l}}_{\text{ATP}}=d_{\text{MT}}$ whereas its maximum is ${\bar{l}}_{\text{ATP}}=d_{\text{MT}}/2$ for inch-worm stepping.

We measure the motor efficiency as the average work against the external load *F* per hydrolysis of one ATP molecule

$$\begin{array}{c} W_{\text{ATP}}=F{\bar{l}}_{\text{ATP}}. \end{array}$$ (12)

*W*_ATP_ is zero both for an unloaded (*F* = 0) and for a stalled motor (*v* = 0). Optimal efficiency is reached at an intermediate force.

### Minimal conditions for directional stepping

We start our discussion by the minimal requirements for directional stepping. In the case of symmetric distance parameters *δ*_+_ = *δ*_−_, the detachment rates of the lead and the trail head are always equal, $k_{i}^{L}=k_{i}^{T}$. The same holds for the binding rates, $k_{i}^{F}=k_{i}^{B}$. Inserting these equalities into the matrix K shows that the solution of Eq (8) fulfills *c*_6_ = *c*_7_. With these symmetries, Eq (9) gives *v* = 0. The symmetry argument remains valid if the power- and recovery stroke rate are load dependent, i.e., when $k_{\text{PS}}^{0}$ and $k_{\text{RS}}^{0}$ differ between the two heads and between states 1, 2, 3 and 4. Despite an asymmetric power stroke, a motor which neither has a coupling between the ATPase state and the binding affinity nor asymmetric force-induced unbinding can not generate directed motion.

With asymmetric force-induced unbinding the rates $k_{i}^{L}$ and $k_{i}^{T}$ are no longer equal but the principle of detailed balance, Eq (4), implies $k_{i}^{F}/k_{i}^{B}=k_{i}^{L}/k_{i}^{T}$. As long as the internal tension *F*_int,*i*_ is independent of state *i* the motor naturally does not move. But if the power strokes in the two heads modulate the tension, directional motility emerges, as will be discussed below.

### Stepping efficiency

In the following we study how the stepping efficiency depends on the kinetic rates and the two distance parameters of force-induced unbinding.

Because the states *M* − *M* and M* − M* have the same elastic energy (their elastic extension is *d*_1_ = *d*_4_ = *d*_MT_), there is an additional symmetry in the master equation, i.e., with respect to the exchange of the hydrolysis rates $k_{\text{RS}}^{0}↔k_{\text{PS}}^{0}$. Therefore the resulting stepping characteristics are symmetric upon inversion of the ratio $k_{\text{PS}}^{0}/k_{\text{RS}}^{0}$, which we use as a dimensionless parameter, along with $k_{-\text{MT}}^{0}/\left(k_{\text{PS}}^{0}+k_{\text{RS}}^{0}\right)$ and $k_{+\text{MT}}^{0}/\left(k_{\text{PS}}^{0}+k_{\text{RS}}^{0}\right)$. From this symmetry we expect that the motor will reach optimal efficiency when $k_{\text{PS}}^{0}/k_{\text{RS}}^{0}=1$.

An increase in the MT binding rate $k_{+\text{MT}}^{0}$ prevents the motor from performing futile ATP hydrolysis cycles in the MT unbound state and, thus, leads to an increased velocity and efficiency (it also increases the processivity, which is not subject of this study), but does not affect the forward/backward stepping probabilities. Thus, we can restrict our search and discussion of the optimal working regime to the high MT affinity limit, i.e., $k_{+\text{MT}}^{0}\gg k_{-\text{MT}}^{0}$. The rates $k_{+\text{MT}}^{0}$ and $k_{-\text{MT}}^{0}$ for the dynein motor approach this limit.

Considering all this, for dynein the number of essential model parameters is reduced to four: the ratio of the hydrolysis rates $k_{\text{PS}}^{0}/k_{\text{RS}}^{0}$, the reduced unbinding rate $k_{-\text{MT}}^{0}/\left(k_{\text{PS}}^{0}+k_{\text{RS}}^{0}\right)$, and the two parameters of the force-induced unbinding, $\bar{\delta }$ and *δ*_*a*_. We study the role of these parameters for the efficient stepping in what follows.

Because the power- and recovery stroke are equally likely to take place in the lead and the trail head, half of the hydrolysis events are inevitably futile and the maximum value the average move per ATP can achieve is *d*_MT_/2 = 4nm or half the value of an ideally coordinated motor. Additional factors that reduce stepping efficiency include further futile cycles if force induced unbinding rate is slower than the hydrolysis cycle and lead head detachment in the case of insufficient asymmetry.

**Fig 4:**
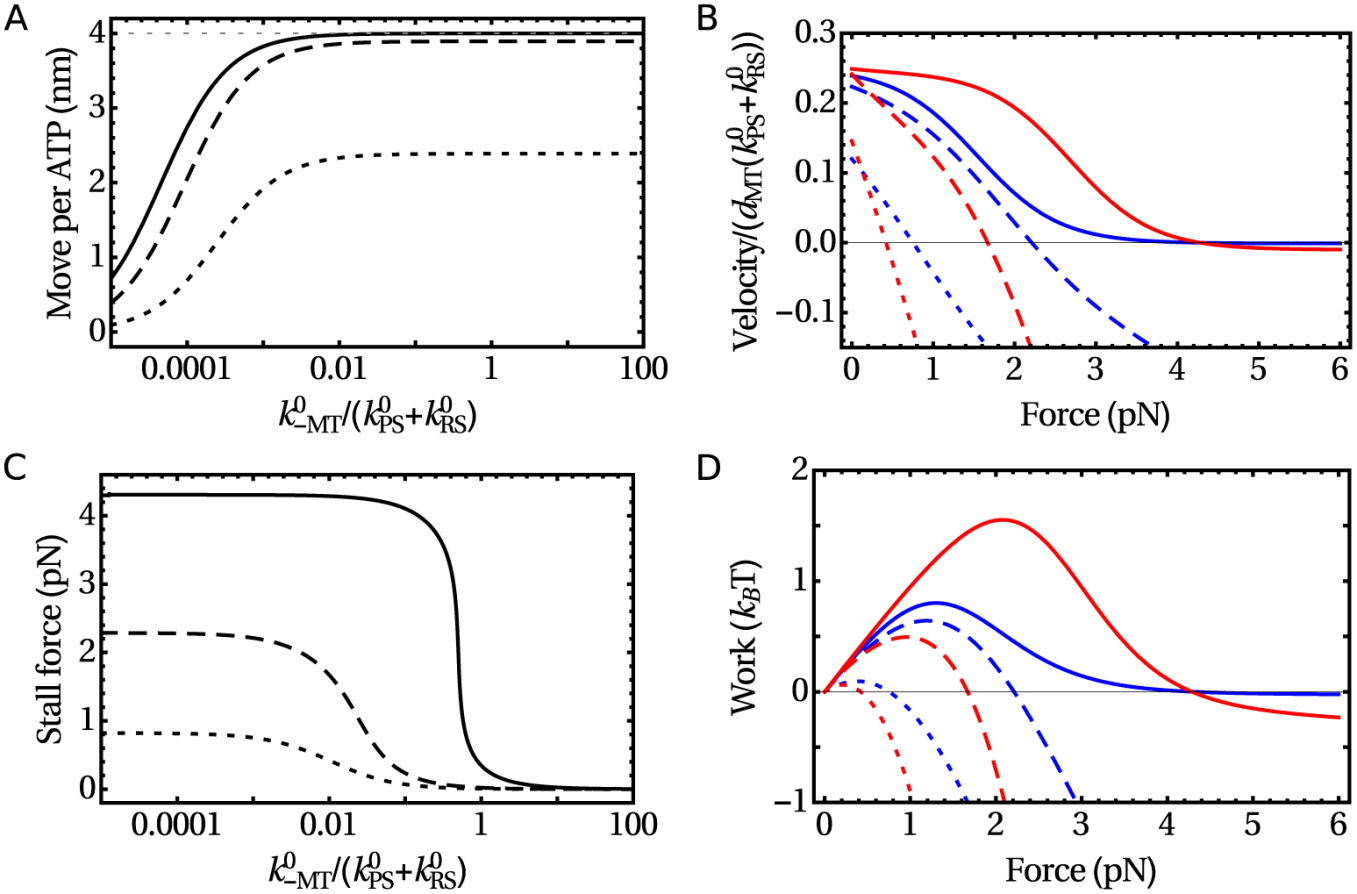
Motor properties in the high MT affinity regime with $k_{\text{PS}}^{0}/k_{\text{RS}}^{0}=1$. (A) The average motor displacement per hydrolysis of one ATP molecule of an unloaded motor as a function of the kinetic rate $k_{-\text{MT}}^{0}$ for different values of the asymmetry parameter. (B) The force–velocity diagram for two values of the kinetic rate $k_{-\text{MT}}^{0}$, $k_{-\text{MT}}^{0}/\left(k_{\text{RS}}^{0}+k_{\text{PS}}^{0}\right)=0.001$ (blue) or 0.01 (red). (C) The stall force as a function of $k_{-\text{MT}}^{0}/\left(k_{\text{RS}}^{0}+k_{\text{PS}}^{0}\right)$. (D) The force dependence of the work produced by the motor for parameters as in (B). In all plots $\delta_{a}/\bar{\delta }=0.1$ (dotted line), 0.33 (dashed line), and 1.0 (solid line).

The average move the motor makes per hydrolysis of one ATP molecule is shown in Fig 4A. The increasing asymmetry improves the stepping efficiency by reducing the probability of lead head detachment and by increasing the trail head detachment rate. As long as the high MT affinity limit ($k_{+\text{MT}}^{0}\gg k_{-\text{MT}}^{0}$) is valid an increasing MT release rate also leads to higher efficiency because it prevents the occurrence of additional hydrolysis events during one step. Beyond that limit, for $k_{-\text{MT}}^{0}$ approaching $k_{+\text{MT}}^{0}$ (not shown) the average move per ATP would decrease due to futile power strokes in the detached head and eventually the processivity would also be strongly reduced.

When subject to an external force in the direction opposing the stepping, the force acting on the lead head increases and that on the trail head decreases, which reduces the asymmetry of force-induced unbinding rates. Thus, the average step length decreases with force and the velocity changes its direction above the stall force. The force–velocity diagram for different values of the reduced unbinding rate $k_{-\text{MT}}^{0}/\left(k_{\text{RS}}^{0}+k_{\text{PS}}^{0}\right)$ and the asymmetry parameter *δ*_*a*_ is plotted in Fig 4B and the stall force as a function of the reduced unbinding rate in Fig 4C. One can notice that the shape of the force–velocity diagram changes with *δ*_*a*_. Different shapes correspond to different regimes of how the load dependence is split between the transition rates which govern forward and backward steps [37]. For moderately asymmetric unbinding rates the load dependence of the transition rates is shared between the two heads and there is a symmetry between the forward and backward movement. However, for $\delta_{a}\to \bar{\delta }$ (*δ*_+_ → 0), only the forward rates become load dependent and the motor behaves as a ratchet, which can be moved forward by an assisting load, but not backward by a resisting. A typical force dependence of the average work generated per ATP molecule is shown in Fig 4D.

**Fig 5:**
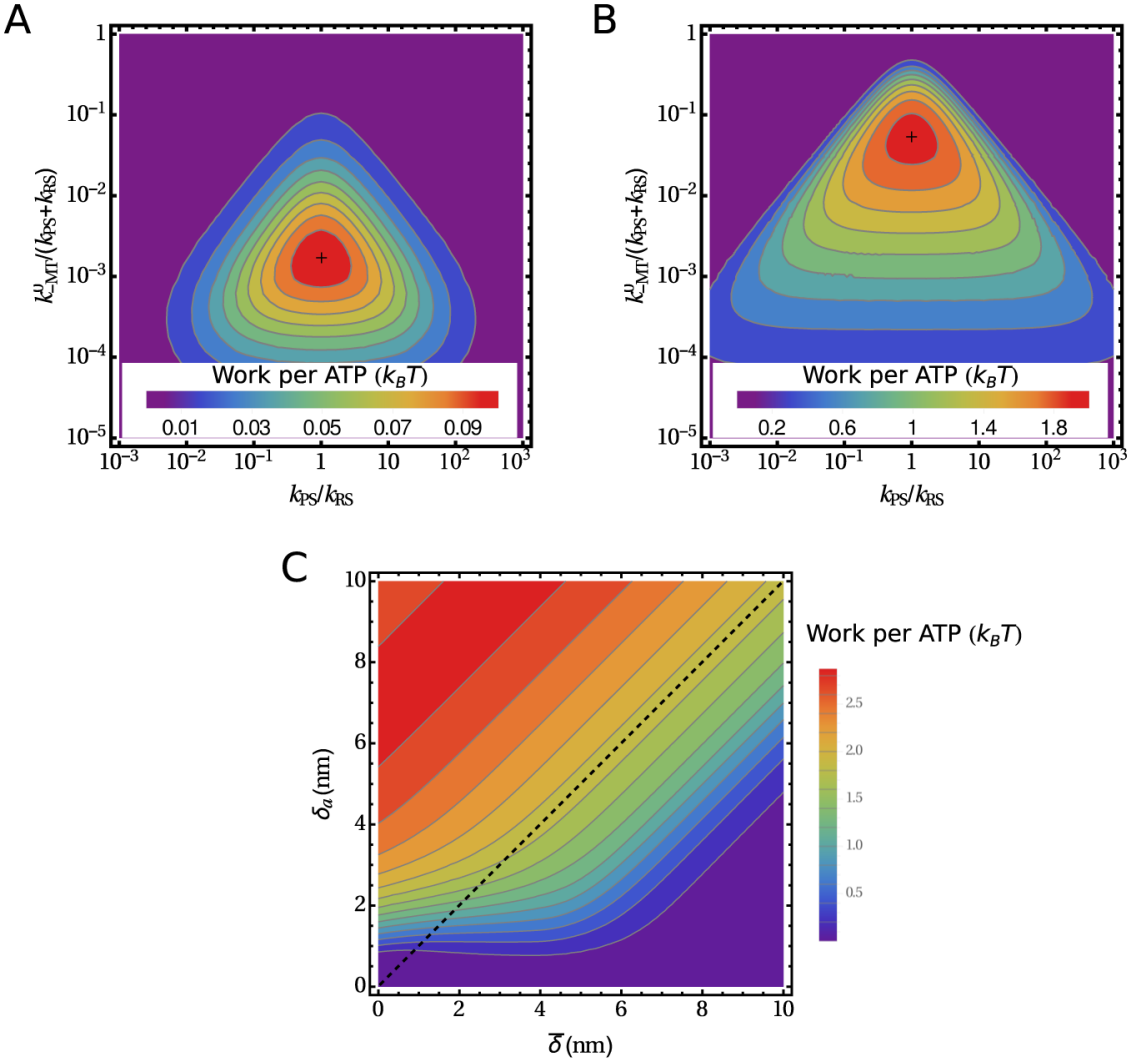
The maximum work per ATP produced by the motor. (A,B) The maximum work per ATP as a function of two dimensionless kinetic parameters for two levels of asymmetry in the force-induced unbinding rates: (A) $\bar{\delta }=6\;\text{nm}$ and $\delta_{a}=0.1\;\bar{\delta }$ and (B) $\bar{\delta }=\delta_{a}=6\;\text{nm}$. In each plot, the maximum is marked by a cross. (C) The work per ATP in the optimal kinetic regime as a function of the mean distance parameter $\bar{\delta }$ and its asymmetry distance *δ*_*a*_. The dashed line separates different bond types with respect to their behavior under backward load: The area below the dashed line describes a slip bond, the area above the line a catch bond, and the line itself an ideal bond.

Fig 5A and 5B shows the maximum work per ATP molecule as a function of kinetic rates. It depends strongly on the asymmetry of the force-induced unbinding as it scales approximately with $\delta_{a}^{2}$. Because of the symmetry discussed above, the maximum work is highest when $k_{\text{PS}}^{0}/k_{\text{RS}}^{0}=1$. Finally, the maximum work per ATP as a function of $\bar{\delta }$ and *δ*_*a*_, while all other parameters have their optimal values, is shown in Fig 5C.

### Model with reversible transitions

**Fig 6:**
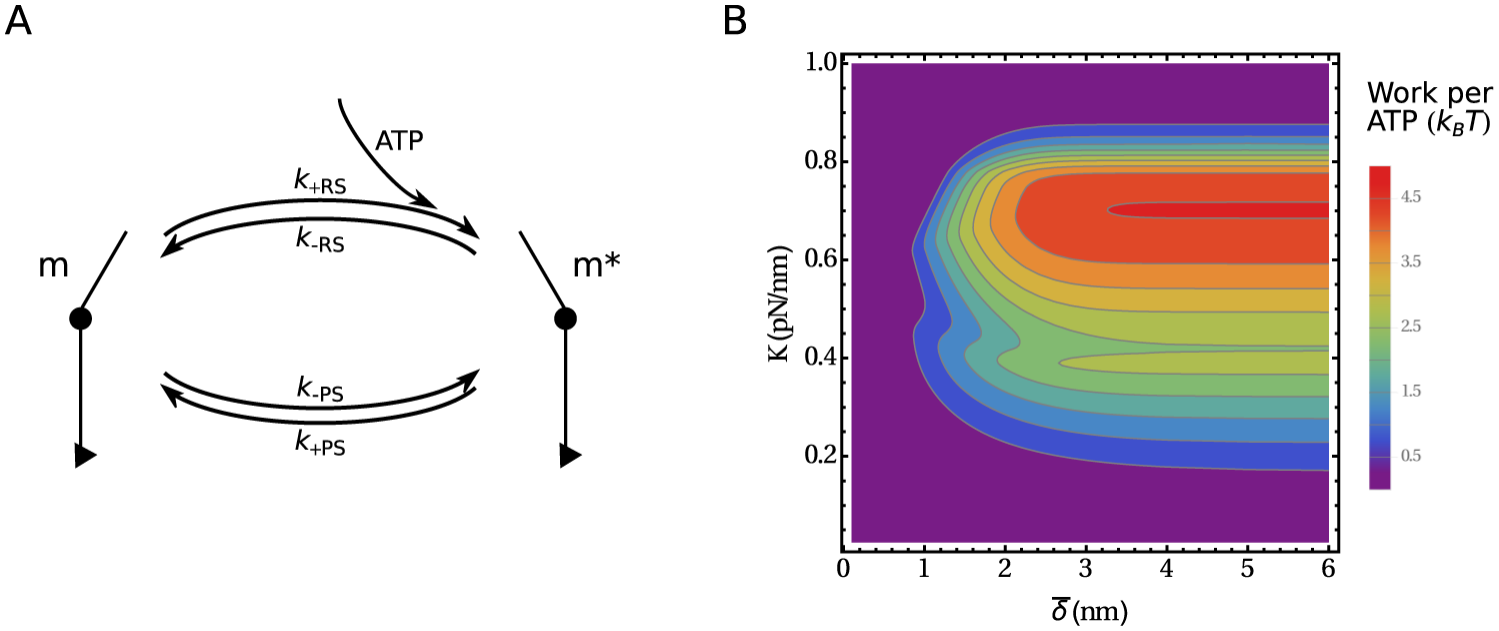
Reversible model. (A) The model includes the reverse recovery stroke (*k*_-RS_) and the reverse power stroke (*k*_-PS_). (B) The maximum work per ATP as a function of the distance parameter $\bar{\delta }$ ($\delta_{a}\equiv \bar{\delta }$) and the connector stiffness *K* while other parameters have the optimal values.

In order to test the validity of the model with load-independent effective rates of the power stroke and the recovery stroke, $k_{\text{RS}}^{0}$ and $k_{\text{PS}}^{0}$, we extend it by incorporating reverse transitions in a way that make the model thermodynamically consistent. Note that even though both transitions take place between the same states in the simplified model, $k_{\text{PS}}^{0}$ is not the reversal of $k_{\text{RS}}^{0}$. A cycle consisting of both returns the head to the original state, but leads to the hydrolysis of one ATP molecule. If we include the reverse rates *k*_−PS_ and *k*_−RS_ (Fig 6A), their ratio fulfills the detailed balance condition in the absence of load

$$\begin{array}{c} \frac{k_{+\text{RS}}^{0}}{k_{-\text{RS}}^{0}}={\text{e}}^{-\frac{\Delta G_{\text{RS}}}{k_{B}T}}\;\text{and}\;\frac{k_{+\text{PS}}^{0}}{k_{-\text{PS}}^{0}}={\text{e}}^{-\frac{\Delta G_{\text{PS}}}{k_{B}T}}, \end{array}$$ (13)

where Δ*G*_RS_ + Δ*G*_PS_ = Δ*G*_ATP_ is the free energy difference of ATP hydrolysis. For physiological ATP, ADP and Pi concentrations its value is about Δ*G*_ATP_ = −25*k*_B_*T*. We distribute it equally between both strokes, Δ*G*_RS_ = Δ*G*_PS_ = Δ*G*_ATP_/2.

In a dimeric molecule or under external load the kinetic rates are influenced by the mechanical strain. The principle of detailed balance states that *k*_+*i*_/*k*_−*i*_ = e^−(Δ*G*_+*i*_ + Δ*U*_+*i*_)/*k*_B_*T*^. We further assume that the load only affects the uphill transition:

$$\begin{array}{cccccc} k_{+i} & =k_{+i}^{0}, & k_{-i} & =k_{+i}^{0}{\text{e}}^{\left(\Delta G_{+i}+\Delta U_{+i}\right)/k_{\text{B}}T} & \text{for}\;\Delta G_{+i}+\Delta U_{+i} & <0 \end{array}$$ (14)

$$\begin{array}{cccccc} k_{-i} & =k_{+i}^{0}, & k_{+i} & =k_{+i}^{0}{\text{e}}^{-\left(\Delta G_{+i}+\Delta U_{+i}\right)/k_{\text{B}}T} & \text{for}\;\Delta G_{+i}+\Delta U_{+i} & >0\;. \end{array}$$ (15)

In other words, the downhill transition is of Eyring type [1], in line with the Leffler-Hammond postulate [38] which implies that the transition point is closer to the state with the higher free energy. A transition point away from the initial state would also lead to a reduced stepping efficiency.

To solve the model, we replace the rates in the matrix K, Eq (7), with the effective ones, $k_{\text{RS}}^{\text{eff}}=k_{+\text{RS}}+k_{-\text{PS}}$ and $k_{\text{PS}}^{\text{eff}}=k_{+\text{PS}}+k_{-\text{RS}}$. The ATPase rate is then calculated as

$$\begin{array}{ccc} r_{\text{ATP}} & = & k_{+\text{RS}}^{0}\left(2c_{1}+c_{2}+c_{3}+2c_{5}+c_{6}+c_{7}\right)-k_{-\text{RS}}^{T}c_{3}-k_{-\text{RS}}^{\prime T}c_{4}\\ & & -k_{-\text{RS}}^{L}c_{2}-k_{-\text{RS}}^{\prime L}c_{4}-k_{-\text{RS}}^{S}\left(c_{7}+c_{8}\right)-k_{-\text{RS}}^{0}\left(c_{6}+c_{8}\right), \end{array}$$ (16)

where $k_{-\text{RS}}^{T/L/S}$ is the reverse recovery stroke rate of the trailing/leading or single bound head. The model becomes equivalent to the simplified (irreversible) one if |Δ*U*| ≪ |Δ*G*_ATP_/2|.

The work of a motor produced per ATP as a function of $\bar{\delta }$ and *K* is shown in Fig 6B. We use $\delta_{a}=\bar{\delta }$, the maximal distance parameter asymmetry outside the “catch bond” regime. The efficiency as a function of $\bar{\delta }$ increases up to $\bar{\delta }∼3\;\text{nm}$ when it reaches a plateau. An increasing connector stiffness *K* first improves the efficiency by increasing the internal forces and consequently the unbinding asymmetry. However, for higher stiffnesses the highly strained state 3 (Fig 3) becomes inaccessible and the motor runs through futile cycles. The efficiency reaches its maximum at around *K* = 0.7pN/nm. The maximum work per ATP is then 4.5*k*_B_*T*, corresponding to an efficiency of 18%.

## Conclusion

Our results show that a dimeric motor protein can generate directed motility if the ATPase cycle is coupled to a lever arm movement and in addition at least one of the two conditions is fulfilled: (i) there is an allosteric interaction between the ATPase state and the track binding affinity, or (ii) the force-induced unbinding rates of its heads from the track are asymmetric. This asymmetry can be rooted directly in the interaction potential between the motor and the track (as proposed in early ratchet models [6]). But it is also possible that strain induces internal conformational changes in the motor which in turn modulate the binding affinity for the track.

Mechanism (i) is well known and proven to be present in kinesins [39], myosins [40] and dyneins [8]. In the context of dynein, we have previously discussed a theoretical model based on this mechanism and shown that it can explain coordinated stepping with a high efficiency [41]. Mechanism (ii), studied in this paper, is less efficient, but proves that a motor like dynein could be functional even without any allosteric interaction through the stalk. This finding should be testable in dynein where the spatial distance between the catalytic sites and the track binding made it possible to interfere with the allosteric coupling [14]. By locking the helices in a state with a high MT binding affinity and at the same time high ATPase rate, the motor should be functional by mechanism (ii) alone. To some extent, the functionality of this mechanism was already demonstrated in heterodimeric motors with one inactive head [20], which were still motile. Physiologically, the lower efficiency of mechanism (ii) provides a possible explanation for the universal presence of two-way coupling between the ATPase cycle, the working stroke and the track binding affinity in linear motor proteins (mechanism (i)). At the same time, asymmetric unbinding could additionally improve the stepping efficiency of dynein, especially at high forces [36]. Interestingly, the opposite effect has been observed in myosin V, which slips backwards under strong loads, but is able to resist forces in the direction of motion [42]. The difference could be related to different physiological functions of both motors, but the requirements on motors performing different functions in the cytoskeleton are still largely unknown. Finally, whereas motor proteins have evolved to work with a high efficiency, the design of artificial bipedal motors is still in its early stage and the focus is on making them move, rather than making them move efficiently. In this respect, asymmetric unbinding can provide a simple and viable mechanism.
